# Effect of omega-3 polyunsaturated fatty acid supplementation on central arterial stiffness and arterial wave reflections in young and older healthy adults

**DOI:** 10.14814/phy2.12438

**Published:** 2015-06-24

**Authors:** Kevin D Monahan, Robert P Feehan, Cheryl Blaha, Daniel J McLaughlin

**Affiliations:** Penn State Hershey Heart and Vascular Institute, Pennsylvania State University College of MedicineHershey, Pennsylvania

**Keywords:** Aging, cardiovascular disease, diet, fish oil, vascular stiffness

## Abstract

Increased central arterial stiffness and enhanced arterial wave reflections may contribute to increased risk of cardiovascular disease development with advancing age. Omega-3 polyunsaturated fatty acid (*n*-3) ingestion may reduce cardiovascular risk via favorable effects exerted on arterial structure and function. We determined the effects of *n*-3 supplementation (4 g/day for 12 weeks) on important measures of central arterial stiffness (carotid-femoral pulse wave velocity; PWV) and arterial wave reflection (central augmentation index) in young (*n* = 12; 25 ± 1-year-old, mean ± SE) and older (*n* = 12; 66 ± 2) healthy adults. We hypothesized that *n*-3 supplementation would decrease carotid-femoral PWV and central augmentation index in older adults. Our results indicate that carotid-femoral PWV and central augmentation index were greater in older (988 ± 65 cm/sec and 33 ± 2%) than in young adults (656 ± 16 cm/sec and 3 ± 4%: both *P* < 0.05 compared to older) before the intervention (Pre). *N*-3 supplementation decreased carotid-femoral PWV in older (Δ-9 ± 2% Precompared to Post; *P* < 0.05), but not young adults (Δ2 ± 3%). Central augmentation index was unchanged by *n*-3 supplementation in young (3 ± 4 vs. 0 ± 4% for Pre and Post, respectively) and older adults (33 ± 2 vs. 35 ± 3%). Arterial blood pressure at rest, although increased with age, was not altered by *n*-3 supplementation in young or older adults. Collectively, these data indicate that 12 weeks of daily *n*-3 supplementation decreases an important measure of central arterial stiffness (carotid-femoral PWV) in older, but not young healthy adults. The mechanism underlying decreased central arterial stiffness with *n*-3 supplementation is unknown, but appears to be independent of effects on arterial blood pressure or arterial wave reflections.

## Introduction

Cardiovascular disease (CVD) is the leading cause of morbidity and mortality worldwide (World Health Organization, 2011). The incidence of CVD increases dramatically with advancing age, such that more than half of all individuals with CVD are over the age of 60 (Go et al. 2014). Thus, it is not surprising that aging has emerged as a prominent risk factor for CVD (Fleg et al. 1990; Wolf and Lewis 1993; Burt et al. 1995). In fact, aging may be the primary risk factor for CVD (Lakatta and Levy 2003a). As the general population ages there is an increased need to better understand the processes by which aging contributes to the development of CVD, as well as to identify effective strategies to prevent or reverse the development of CVD with age.

Numerous changes in cardiovascular system structure and function likely contribute to the increased prevalence of CVD with advancing age (Lakatta 1993, 2002, 2003; Lakatta and Levy 2003a,b). Specifically, the notion that ‘arterial aging’ contributes significantly to CVD development with age is an important emerging concept (Lakatta 2002; Lakatta and Levy 2003a; Seals 2014). Effects of arterial aging can be observed by examination of indices providing insight into arterial stiffness, such as carotid-femoral pulse wave velocity (PWV), as well as integrative indices of arterial wave reflections, such as central augmentation index. In this context, both carotid-femoral PWV and central augmentation index increase with advancing age (Avolio et al. 1985; Smulyan et al. 2001; Mitchell et al. 2004; McEniery et al. 2005) and possess prognostic capabilities (Meaume et al. 2001a,b; Sutton-Tyrrell et al. 2005; Willum-Hansen et al. 2006; Mitchell et al. 2010) over that provided by conventional cardiovascular risk factors (Sutton-Tyrrell et al. 2005; Cecelja and Chowienczyk 2009; Mitchell et al. 2010; Ben-Shlomo et al. 2014). Thus, such measures likely can be used as biomarkers and targets for interventions or therapies aimed at reducing the burden of CVD (Vlachopoulos et al. 2010a,b).

Dietary intake of omega-3 polyunsaturated fatty acids (*n*-3s), such as eicosapentaenoic acid and docosahexanoic acid, is inversely associated with rates of cardiovascular-related morbidity and mortality (Bang et al. 1976; Kromhout et al. 1985; Siscovick et al. 1995; Daviglus et al. 1997; Albert et al. 1998; He et al. 2004). The specific mechanisms underlying the cardioprotective effect of *n*-3s is likely multifactorial, possibly affecting physiological measures such as arterial blood pressure (BP) (Mano et al. 1995), inflammation (Pischon et al. 2003), thrombosis and hemostasis (Knapp 1997), cardiac electrical stability (Leaf et al. 2003), blood lipids (Harris 1997), and arterial function (Mano et al. 1995; Goode et al. 1997). In regard to arterial function, observational studies report decreased arterial stiffness in individuals habitually consuming larger quantities of fish (Hamazaki et al. 1988; Yamada et al. 2000), a rich source of *n*-3s. Subsequent interventional trials supported a beneficial effect of *n*-3 supplementation on measures of arterial stiffness, such as PWV (Tomiyama et al. 2005; Mita et al. 2007; Satoh et al. 2009) and arterial wave reflections (Iketani et al. 2013; Siasos et al. 2013). However, it is presently unknown if increased dietary intake of *n*-3s beneficially affects detrimental age-associated changes in central arterial stiffness and arterial wave reflections in healthy adults.

Accordingly, the purpose of the present study was to determine if *n*-3 supplementation improves measures of central arterial stiffness and arterial wave reflections in healthy young and older adults. We hypothesized that 12 weeks of dietary *n*-3 supplementation would decrease central arterial stiffness and reduce effects of arterial wave reflections in healthy older adults, as a result of detrimental age-associated changes. Such effects could help establish a therapeutic potential for *n*-3 supplementation in older adults, via effects on emerging biomarkers of arterial aging.

## Materials and Methods

### Subjects

Twelve young and 12 older healthy adults participated in this study. Inclusion criteria were: age 21–35 (young) or 60–80 (older), healthy (as assessed by review of medical history and physical examination), brachial BP at rest <140/90 mmHg, nonsmoker, nonobese (body mass index <30 kg/m^2^), total cholesterol <6.2 mmol/L, triglycerides <2.1 mmol/L, and unmedicated. The Pennsylvania State University College of Medicine Institutional Review Board approved the study. All subjects provided signed informed consent prior to testing.

### Experimental protocol

Subjects did not consume alcohol (24 h), caffeine (12 h), or food (12 h) before the experiments. Prior to obtaining baseline measurements (Pre) subjects rested on an examination table in a quiet dimly lit room for 30 min. After obtaining Premeasures, *n*-3 supplementation was begun and continued daily for 12 weeks. Subsequently, the Premeasurements were repeated (Post) in all subjects under identical experimental/laboratory conditions.

#### *n*-3 supplementation

Subjects were instructed to ingest four *n*-3 containing capsules daily, in two divided doses, for 12 weeks. Each capsule contained 1000 mg of *n*-3s [465 mg eicosapentaenoic acid and 375 mg docosahexanoic acid; Lovaza®, GlaxoSmithKline]. Compliance to supplementation was assessed in all subjects by pill diary, pill count, and weekly phone calls to subjects as well as in a subgroup or young and older subjects by quantification of erythrocyte eicosapentaenoic acid and docosahexanoic acid content (see below).

### Measurements

#### BP and heart rate

A semiautomated device (Dinamap; GE Medical system) was used to measure BP over the brachial artery. Heart rate was determined via electrocardiogram.

#### PWV

Central and peripheral arterial stiffness are reported as PWV. PWV quantifies the velocity of the forward traveling arterial pressure wave through an arterial segment based on the measured time delay between arrival of the systolic upstroke at proximal and distal arterial sites (Bramwell and Hill 1922). Two nondirectional Doppler flow probes (Model 810 A; Parks Medical Electronics, Inc., Aloha, OR) were used to simultaneously record continuous blood flow signals at the sites. The mean time delay (Δ time) between arrival of systolic upstrokes (initial sharp upstroke after the R-wave) was later computed over 6–15 cardiac cycles (Chart, ADInstruments). Straight path distances, between the two recording sites, were measured with a cloth tape and PWV was calculated as (Δdistance/Δtime) (Hess et al. 2009). PWV was measured in central (carotid-femoral) and peripheral (arm and leg) arterial segments. Common carotid and common femoral artery recording sites were used to determine carotid-femoral PWV, brachial, and radial artery recording sites were used to determine arm PWV, and common femoral and posterior tibial artery recording sites were used to determine leg PWV.

#### Arterial tonometry

Arterial tonometry was performed transcutaneously over the right radial artery. Recorded radial artery waveforms were calibrated by synthesizing a central (aortic) pressure waveform using a generalized transfer function (Sphygmocor, AtCor Medical) (Gallagher et al. 2004). Both systolic and diastolic aortic BP was derived using this technique. The synthesized central aortic waveform was used to quantify central augmentation index as the percent increase in the peak systolic waveform from the systolic shoulder. Central augmentation index was also calculated at a standardized heart rate of 75 beats/min.

#### Blood biochemistry

Eicosapentaenoic acid and docosahexanoic acid content of erythrocytes were determined in a subgroup of young (*n* = 8) and older subjects (*n* = 8), using established laboratory methodologies (Harris et al. 2012). Other biochemical parameters were obtained using standard laboratory methods by our hospital's clinical laboratory.

### Statistical analysis

Differences in baseline subject characteristics were determined by *t*-test and repeated measures ANOVA was used to determine effects of the intervention. Specific contrasts were made using Newman–Keuls post hoc tests. Statistical significance was established at *P* < 0.05. All data are presented as mean ± SE.

## Results

### Subject characteristics

Subject characteristics before (Pre) and after (Post) *n*-3 supplementation are presented in Table1. Older subjects were of shorter stature and lower body mass than the young, although body mass index was similar in the two groups. Blood lipids including total, LDL, and HDL cholesterol, as well as triglycerides were greater (*P* < 0.05) in older as compared to young adults. The only one of these variables to change after *n*-3 supplementation was triglycerides, which decreased (*P* < 0.05) in older adults. C-reactive protein levels were similar in young and older adults (Pre) and decreased significantly with *n*-3 supplementation in the subject population as a whole (i.e., in young and older subjects combined), but not in the individual groups (i.e., not in the young or older groups individually). Eicosapentaenoic acid and docosahexanoic acid content of erythrocytes was similar in young and older subjects before supplementation (Pre) and increased with supplementation (Post; *P* < 0.05 Pre compared to Post) in both young and older subject groups.

**Table 1:** Subject characteristics before (Pre) and after (Post) 12 weeks of daily omega-3 fatty acid supplementation

Variable	Young (*n* = 12)		Older (*n* = 12)	
	Pre	Post	Pre	Post
Sex (m/f)	6/6	–	8/4	–
Age (years)	25 ± 1	–	66 ± 2†	–
Height (cm)	177.6 ± 2.3	177.7 ± 2.1	170.2 ± 2.1†	170.6 ± 2.2†
Weight (Kg)	78.1 ± 4.1	78.7 ± 4.0	71.8 ± 4†	72.2 ± 4†
BMI (Kg/m^2^)	24.6 ± 0.9	24.7 ± 0.9	24.6 ± 0.9	24.6 ± 0.9
HDL cholesterol (mmol/L)	1.3 ± 0.1	1.3 ± 0.1	1.5 ± 0.1†	1.6 ± 0.1†
LDL cholesterol (mmol/L)	2.6 ± 0.2	2.6 ± 0.2	3.0 ± 0.1†	3.1 ± 0.1†
Total cholesterol (mmol/L)	4.2 ± 0.2	4.2 ± 0.2	4.9 ± 0.2†	4.9 ± 0.2†
Triglyceride (mmol/L)	0.79 ± 0.08	0.76 ± 0.09	1.01 ± 0.10†	0.64 ± 0.04*
C-reactive protein (mg/L)	2.9 ± 1.2	1.0 ± 0.2	2.1 ± 0.5	1.2 ± 0.4
Erythrocyte EPA content (%)	0.51 ± 0.04	1.99 ± 0.39*	0.61 ± 0.07	3.71 ± 0.34*†
Erythrocyte DHA content (%)	4.11 ± 0.24	6.35 ± 0.55*	3.92 ± 0.36	7.68 ± 0.20*†

BMI, body mass index

HDL, high-density lipoprotein

LDL, low-density lipoprotein

EPA, eicosapentaenoic acid

DHA, docosahexanoic acid.

* *P* *<* 0.05 vs. Pre (same age group).

† *P* < 0.05 vs. young (same time point).

All values are means ± SE.

Brachial systolic, diastolic, and mean BP, as well as heart rate at rest, were greater in older as compared to young adults (Pre). None of these variables was altered by *n*-3 supplementation. Carotid systolic and diastolic BP displayed similar patterns of response as brachial BP, although increases in carotid systolic BP with age were found to be greater than brachial BP. We found that central augmentation index was greater in older as compared to young adults and was unaffected by *n*-3 supplementation in both groups. These effects on central augmentation index were similar when data were compared at a standardized heart rate of 75 beats/min (Table2).

**Table 2:** Resting hemodynamics before (Pre) and after (Post) 12 weeks of omega-3 fatty acid supplementation

Variable	Young		Older	
	Pre	Post	Pre	Post
Heart rate (beats/min)	55 ± 3	53 ± 3	61 ± 3*	57 ± 2*
Brachial systolic BP (mmHg)	110 ± 3	110 ± 3	119 ± 4*	120 ± 4*
Brachial diastolic BP (mmHg)	62 ± 2	62 ± 1	71 ± 2*	69 ± 1*
Brachial mean BP (mmHg)	85 ± 2	86 ± 2	90 ± 2*	90 ± 2*
Carotid systolic BP (mmHg)	92 ± 2	93 ± 2	112 ± 4*	115 ± 4*
Carotid diastolic BP (mmHg)	63 ± 2	62 ± 2	73 ± 2*	70 ± 1*
AI (%)	3 ± 4	0 ± 4	33 ± 2*	35 ± 3*
AI@_HR75_ (%)	−6 ± 3	−9 ± 4	26 ± 2*	26 ± 3*

BP, blood pressure

AI, central augmentation index

AI@_HR75_, central augmentation index at a standardized heart rate of 75 beats/min.

* *P* < 0.05 vs. young (same time point).

All values are means ± SE.

**Figure 1:**
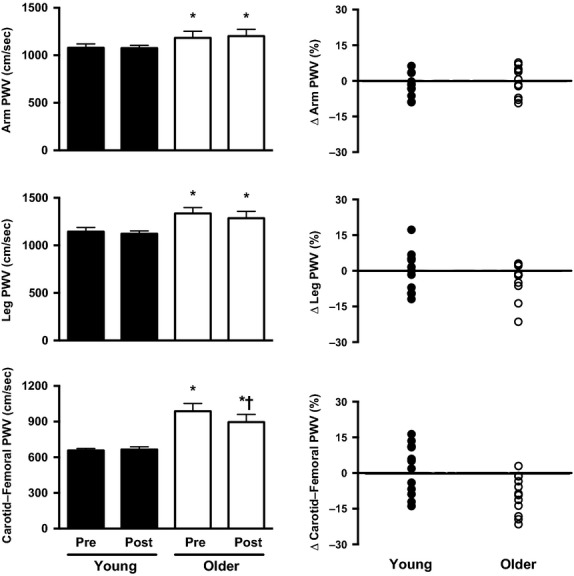
Measures of arterial stiffness (pulse wave velocity; PWV) measured before (Pre) and after (Post) 12 weeks of omega-3 fatty acids (*n*-3) supplementation in young (filled bars/symbols) and older adults (open bars/symbols). Arterial stiffness was measured as arm PWV (upper graphs), leg PWV (middle graphs), and carotid-femoral PWV (lower graphs). Group responses (left panels; mean ± SE; Pre and Post *n*-3 supplementation) as well as the individual subject-by-subject responses (right panels; each symbol represents an individual subject; values are percent change in PWV Post compared to Pre). Results indicate that carotid-femoral PWV was decreased by *n*-3 supplementation in older adults. Values in left panels are mean ± SE. *P *<* 0.05 vs. young (same time point); ^†^*P* < 0.05 vs. Pre (same age group).

PWV was greater in the arm, leg, and aorta of older as compared to young adults before *n*-3 supplementation (Pre). In young adults, *n*-3 supplementation had no effect on PWV of central or peripheral origin. In contrast, *n*-3 supplementation decreased carotid-femoral PWV in older adults (988 ± 65 cm/sec Pre and 895 ± 65 cm/sec Post; *P* < 0.05 Pre compared to Post) (Fig.1); however, levels after *n*-3 supplementation were still greater than in the young (i.e., carotid-femoral PWV was still increased in older compared to young adults after *n*-3 supplementation). Individual responses in PWV (percent change Pre to Post) are also reported on a subject-by-subject basis in Figure1.

## Discussion

The primary new finding of this study is that carotid-femoral PWV, a measure of central arterial stiffness associated with cardiovascular-related morbidity and mortality in humans, is decreased by 12 weeks of dietary *n*-3 supplementation in healthy older adults. Importantly, decreases in carotid-femoral PWV in older adults with *n*-3 supplementation occurred independent of effects on BP or arterial wave reflections. These findings provide support for the concept that increased *n*-3 intake may be an efficacious therapy in the primary prevention of CVD in aging humans through effects on central arterial stiffness.

Measures of central arterial stiffness, such as carotid-femoral PWV, provide a surrogate measure for common cardiovascular endpoints (e.g., cardiovascular-related mortality and risk for cardiovascular events such as myocardial infarction and stroke) in numerous study populations (Blacher et al. 1999a,b; Meaume et al. 2001a; Cruickshank et al. 2002; Laurent et al. 2006; Mattace-Raso et al. 2006; Vlachopoulos et al. 2010b). These associations between carotid-femoral PWV and cardiovascular risk may be the result of increased central arterial stiffness on variables such as cardiac afterload (Nichols and O'Rourke 2005), left ventricular hypertrophy (Toprak et al. 2009), BP (Kaess et al. 2012), and impaired coronary perfusion (Watanabe et al. 1993). Thus, it is not surprising that carotid-femoral PWV has become a frequent target (i.e., intermediary endpoint) for therapies/interventions aimed at reducing cardiovascular risk (Laurent et al. 2006).

Similarly, central hemodynamic measurements associated with enhanced effects of arterial wave reflections provide insight into cardiovascular risk (Vlachopoulos et al. 2010a). In numerous populations, measures such as central augmentation index (London et al. 2001; Ueda et al. 2004; Weber et al. 2005; Vlachopoulos et al. 2010a) and aortic systolic BP (Lu et al. 2001; Safar et al. 2002; Pini et al. 2008; Vlachopoulos et al. 2010a) possess prognostic capabilities. However, in our study these measures of vascular health/function were unaffected by *n*-3 supplementation in both young and older adults. This finding may be surprising based upon the fact that a reduced velocity of the forward (incident) pulse wave through the aorta would be expected to delay the arrival of the reflected wave in the proximal aorta (Nichols and O'Rourke 2005). However, our data do not support this as we saw clear effects of *n*-3 supplementation on carotid-femoral PWV with no detectable effect on measures of the central pulse wave, such as central systolic BP and augmentation index. This finding is consistent with the observation that various factors (i.e., aging, diabetes, and metabolic syndrome) may differentially affect measures of carotid-femoral PWV and central augmentation index (Lacy et al. 2004; McEniery et al. 2005; Tousoulis et al. 2014), suggesting that both measures may provide independent insight into varying aspects of vascular health and function.

Prior studies have addressed the effect that *n*-3 supplementation may exert on measures of central arterial stiffness and arterial wave reflections in humans. These studies have produced conflicting results with some reporting decreased central artery stiffness (Mita et al. 2007; Wang et al. 2008; Anderson et al. 2009; Satoh et al. 2009; Siasos et al. 2013; Tousoulis et al. 2014) and arterial wave reflections (Iketani et al. 2013; Siasos et al. 2013) associated with *n*-3 supplementation or greater dietary intakes of *n*-3s while other studies report no effect on central arterial stiffness (Sanders et al. 2011; Tomiyama et al. 2011; Root et al. 2013; Singhal et al. 2013) or arterial wave reflections (Sanders et al. 2011; Tomiyama et al. 2011; Root et al. 2013; Tousoulis et al. 2014). Differences in the findings between the present study and prior research may be related to differences in study populations (i.e., healthy compared to diseased populations or young compared to older adults), as well as differences in dosing (i.e., lower doses than used in the present study), duration, and relative eicosapentaenoic acid and docosahexanoic acid proportions contained in the *n*-3 supplements. It is not possible for us to determine with certainty if the reduction in the carotid-femoral PWV we observed with *n*-3 supplementation (−9% or −93 cm/sec) is of large enough magnitude to be clinically/physiological significant. However, prior studies suggest that increases in carotid-femoral PWV of 100 cm/sec are associated with a 7–19% relative increase in all-cause or cardiovascular-related mortality, independent of traditional risk factors (Meaume et al. 2001a; Cruickshank et al. 2002; Vlachopoulos et al. 2010b; Ben-Shlomo et al. 2014). Based on the prevalence of CVD in our society such changes in risk would appear to be physiologically significant.

The specific mechanism(s) by which central arterial stiffness (carotid-femoral PWV) is decreased by *n*-3 supplementation in older, but not young healthy adults is unclear. However, there are at least several factors that may contribute. The short duration (12 weeks) over which we observed effects of *n*-3 supplementation on central arterial stiffness might suggest functional rather than structural mechanisms contributed to our observed response. For instance, improved endothelial function may have contributed to reduced carotid-femoral PWV in older adults with *n*-3 supplementation. Previous studies have reported improvements in endothelial function after *n*-3 supplementation (Wang et al. 2012). The factor linking improved endothelial function with arterial stiffness following *n*-3 supplementation may be increased nitric oxide synthesis. Nitric oxide has been suggested to be an important modulator of arterial stiffness (Wilkinson et al. 2002). Alternatively, as decreases in inflammatory biomarkers are associated with decreased arterial stiffness (Yasmin et al. 2004), it is possible that our results may be linked to an anti-inflammatory effect of *n*-3 supplementation. We observed a decrease in C-reactive protein after *n*-3 supplementation in our overall cohort (young and older subject groups combined) suggesting a possible anti-inflammatory effect of *n*-3 supplementation. However, the magnitude of effect was similar in younger and older groups, despite no changes in arterial stiffness in the former. From these findings alone, it is not possible to implicate an anti-inflammatory effect of *n*-3 supplements in altered central arterial stiffness in older adults. Finally it is possible that *n*-3 supplementation decreased basal sympathetic nervous system outflow resulting in decreased central arterial stiffness (Bruno et al. 2012). However, previous data suggest that at least in young healthy adults that basal sympathetic nervous system outflow is not decreased by *n*-3 supplementation (Monahan et al. 2004). We cannot exclude the possibility that basal sympathetic nervous system outflow did not decrease in older healthy adults with *n*-3 supplementation contributing to decreases in central arterial stiffness (carotid-femoral PWV).

Eicosapentaenoic acid and docosahexanoic acid content of erythrocytes increased in both young and older adults with *n*-3 supplementation, although increases were greater in older than in young adults. We are uncertain why this was the case. Subject compliance appeared to be excellent based on subject diaries (kept by the subject), pill counts (performed by the investigators), and weekly reminder calls to subjects by a member of the investigative team. We are not aware of instances in the literature, which provide an explanation for this divergent effect of supplementation in our two groups. This would appear to be an important area for future investigation as incorporation of eicosapentaenoic acid and docosahexanoic acid into the cells of the body is likely to be a critical factor in the health-producing benefits of *n*-3s.

This study has several limitations. First, we studied only healthy adults. Thus, our findings may not be representative of responses in other study populations. Second, this study was not designed or powered to address whether or not responses differed between the sexes. Based on our small sample, it did not appear to. Third, this study was not a randomized controlled trial and all subjects received *n*-3 supplementation in an unblinded fashion. Fourth, our study did not specifically test the role played by the endothelium or in sympathetic nervous system outflow function in the improvements in central arterial stiffness in older adults. Addressing the role of these specific factors would appear to be important in the future. Fifth, we used straight path distances between the carotid artery and femoral artery recording sites to calculate carotid-femoral PWV. This can lead to overestimation of absolute PWV levels compared to other methods (Sugawara et al. 2010). Lastly, the dose of *n*-3 was large and applied for a 12-week period of time. It is not possible to determine what effect would be observed if either of these variables had been applied in a different manner.

## Conclusions

Our findings indicate that 12 weeks of *n*-3 supplementation reduces an important measure of central arterial stiffness (carotid-femoral PWV) in healthy older adults. These decreases were not observed in young adults and occurred independent of effects on either BP or arterial wave reflections. Importantly, these effects occurred over a relatively short period of time. These data provide further support for the concept that *n*-3 supplementation may provide a potent and cost-effective method for primary prevention of age-associated CVD development in humans.

## Conflict of Interest

None of the authors have any conflicts of interest to disclose.
